# Tumour Calcification and Calciphylaxis in End-Stage Renal Disease

**DOI:** 10.1155/2014/108071

**Published:** 2014-11-18

**Authors:** Jia Di, Zhenxing Jiang, Min Yang

**Affiliations:** ^1^Department of Nephrology, The Third Affiliated Hospital of Soochow University, Changzhou 213003, China; ^2^Department of Medical Imaging, The Third Affiliated Hospital of Soochow University, Changzhou 213003, China

## Abstract

Although soft tissue and vascular calcifications are common in CKD and progress as an independent risk factor of all-cause mortality, tumour calcification and calciphylaxis are uncommon in patients with end-stage renal disease (ESRD). Here, we discuss a rare case of a patient with tumour calcification complicated with calciphylaxis developed septic shock from infection. Our patient is a 57-year-old man in his late stage of renal disease who presented with a huge mass at the right hip and necrotic cutaneous ulcers on the lower legs followed by local and systemic infection and death due to septic shock.

## 1. Introduction

Soft tissue and vascular calcifications have been recognized as an important complication of uraemia and focus of studies. Tumoral calcification was first described in 1943 and characterized by one or more periarticular soft tissue calcifications. Calciphylaxis, also known as calcific uremic arteriolopathy (CUA), has been reported to occur in 1% to 4.5% of patients in dialysis [1] and represents a severe complication of end-stage renal disease. Here, we report a rare case of a patient with tumour calcification complicated with calciphylaxis developed septic shock from infection.

## 2. Case Report

**Figure 1:**
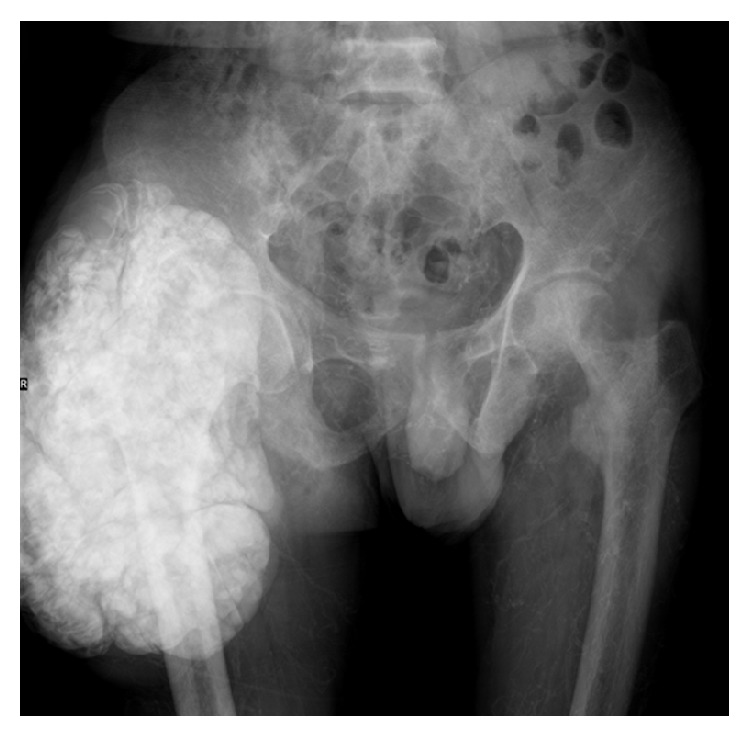
Pelvic X-ray indicated a high density mass, measuring about 26 × 18 cm in size near the right hip joint.

**Figure 2:**
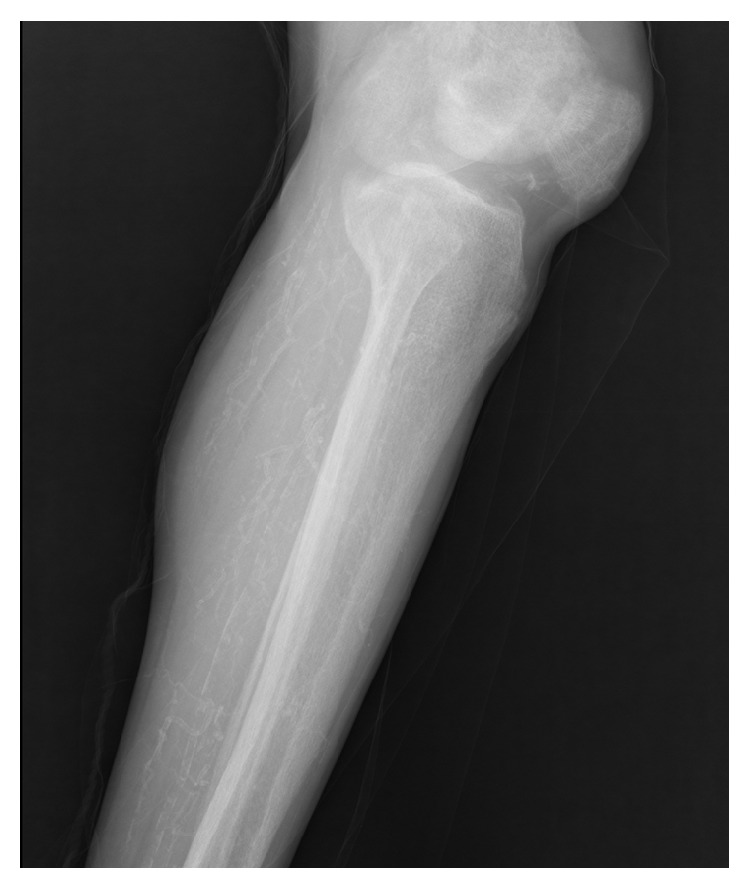
Diffuse small artery calcification of lower legs.

A 57-year-old male patient was admitted to the department of orthopedics because of a painlessly growing mass at the right hip for 4 years. He was diagnosed with ESRD secondary to chronic nephritis since 2002 and received intermittent hemodialysis. Dialysate calcium concentration was 1.50 mmol/L. He had been taking regular alfacalcidol at a dose of 0.25 ug/day for 10 years without follow-up. The physical examination revealed that the mass was about 25 × 20 cm in size at the right hip. Laboratory investigation showed elevated plasma levels of phosphate (10.44 mg/dL) and calcium-phosphate product (78.6 mg^2^/dL^2^). Serum calcium level was normal (7.53 mg/dL) and iPTH was 1375 ng/mL. Hemoglobin was 90.8 g/L, serum urea was 15.93 mmol/L, serum creatinine was 721.1 *μ*mmol/L, and alkaline phosphatase was 752 u/L. Pelvic X-ray indicated a high density mass, measuring about 26 × 18 cm in size near the right hip joint (Figure 1). X-ray indicated diffuse small artery calcification in his lower legs (Figure 2). After admission, alfacalcidol was immediately stopped. Noncalcium-phosphate binders (Fosrenol) and tumor resection were advised but the patient refused because of economic condition and he was discharged from hospital. Within 6 months of discharge, the patient presented with violaceous skin lesions and subcutaneous plaques which were not associated with a history of trauma. Then, he was followed by necrotic ulcers on both lower legs and died few days later due to septic shock.

## 3. Discussion

Tumour calcification complicated with calciphylaxis is rare in end-stage renal disease and is associated with high mortality. We reported a case of tumour calcification and calciphylaxis in a chronic hemodialysis patient with poor outcome. Tumour calcifications are made of massive calcium-phosphate deposits that are usually periarticular in location but do not involve the joints or their capsules [2]. This patient presented a huge tumour calcification which was relatively rare. Review of the literature shows that high calcium-phosphate product, secondary hyperparathyroidism, vitamin D, and aluminium overload are risk factors for development of soft tissue calcification [3]. Our patient presented high phosphate and calcium-phosphate product and elevated iPTH. Besides, long term vitamin D treatment without follow-up was also a risk factor risk factor of his tumour calcification. The treatment of tumour calcifications in dialysis remains usually unsuccessful. In this case, it was inappropriate to suggest that our patient surgically excise the mass because, even amenable to total resection, it will recur unless the biochemical abnormalities are corrected. Parathyroidectomy might be effective to treat our patient; however, soft tissue calcification can occur or worsen even in the absence of severe secondary hyperparathyroidism [4]. Our patient also presented with diffuse small artery calcification of lower limbs as plain radiograph exhibiting a linear or railroad-track arrangement. After discharge, he developed violaceous skin lesions and subcutaneous plaques followed by severe, progressive necrotic ulcers on both lower limbs, which was diagnosed as calciphylaxis. Calciphylaxis represents a life-threatening form of vascular calcification that involves calcium deposition in the walls of small- and medium-sized arteries with consequent skin ischemia and necrosis [5]. Lower extremities are most commonly involved as in the case of our patient. The pathogenesis of CUA is still not well understood but may involve abnormal calcium-phosphate homeostasis. In this case, hyperphosphatemia, elevated calcium-phosphorus product, secondary hyperparathyroidism, and treatment with vitamin D analogs were recognized as risk factors associated with the development of CUA. Gold standard treatment for CUA includes parathyroidectomy, reduction of calcium-phosphate product, cinacalcet, sodium thiosulfate, hyperbaric oxygen, skin grafting, and iloprost infusions [6]. But the mortality rate is still high, with more than 50 percent of patients dying within one year of diagnosis [1]. Our patient died of septic shock quickly after necrotic ulcers despite supporting an antibiotic therapy.
